# A strategy to identify event specific hospitalizations in large health claims databases

**DOI:** 10.1186/s12913-022-08107-x

**Published:** 2022-05-26

**Authors:** Joshua Lambert, Harpal Sandhu, Emily Kean, Teenu Xavier, Aviv Brokman, Zachary Steckler, Lee Park, Arnold Stromberg

**Affiliations:** 1 College of Nursing, University of Cincinnati, Procter Hall, Cincinnati, OH 45219 USA; 2 Department of Bioengineering, University of Louisville Speed School of Engineering, Eastern Pkwy, Louisville, USA; 3 Dr. Bing Zhang Department of Statistics, University of Kentucky, Lexington, USA

**Keywords:** Claims data, Classification, COVID-19, Methodology, COVID-19 research database

## Abstract

**Background:**

Health insurance claims data offer a unique opportunity to study disease distribution on a large scale. Challenges arise in the process of accurately analyzing these raw data. One important challenge to overcome is the accurate classification of study outcomes. For example, using claims data, there is no clear way of classifying hospitalizations due to a specific event. This is because of the inherent disjointedness and lack of context that typically come with raw claims data.

**Methods:**

In this paper, we propose a framework for classifying hospitalizations due to a specific event. We then tested this framework in a private health insurance claims database (Symphony) with approximately 4 million US adults who tested positive with COVID-19 between March and December 2020. Our claims specific COVID-19 related hospitalizations proportion is then compared to nationally reported rates from the Centers for Disease Control by age.

**Results:**

Across all ages (18 +) the total percentage of Symphony patients who met our definition of hospitalized due to COVID-19 was 7.3% which was similar to the CDC’s estimate of 7.5%. By age group, defined by the CDC, our estimates vs. the CDC’s estimates were 18–49: 2.7% vs. 3%, 50–64: 8.2% vs. 9.2%, and 65 + : 14.6% vs. 28.1%.

**Conclusions:**

The proposed methodology is a rigorous way to define event specific hospitalizations in claims data. This methodology can be extended to many different types of events and used on a variety of different types of claims databases.

## Background

Prescription and health insurance claims providers can deliver unique patient-level retail pharmacy, diagnosis, and procedure data. These data can range in size and complexity depending on the provider [1–3]. Successfully using these data in medical research is not an easy task and requires some key considerations [1]. Some of this difficulty comes from the lack of structure and context to how certain International Classification of Diseases (ICD)-10, Current Procedural Terminology (CPT), or drug codes are grouped with one another around an event of interest. For example, a hospitalization CPT code does not link to the diagnosis code that caused it, nor does the drug code that was prescribed because of the event. This disjointness is a major hurdle for researchers hoping to harness these large claims data for their research question of interest.

### Motivation

These issues arose in our own research, where we sought to use a large healthcare claims database called Symphony. Like many others, Symphony Health Solutions includes data on retail pharmacy claims, medical claims, and readmittance claims.

Symphony Health is a leading provider of high-value data for biopharmaceutical manufacturers, healthcare providers, and payers. The company helps clients understand disease incidence, prevalence, progression, treatment, and influences along the patient and prescriber journeys by connecting and integrating a broad set of primary and secondary data. Symphony Health derived data improves health management decisions, and helps clients drive revenue growth while providing critical insights on how to effectively adapt to the changing healthcare ecosystem.

For each diagnosis, procedure, or prescription event, Symphony provided us with a patient ID, relevant code (e.g., ICD-10 code), and date of the event. However, it was not known whether a diagnosis for a patient is a primary, secondary, or subsequent diagnosis. No range or specific information about the date of the event was provided (example: no range of when hospitalization, or procedure occurred. Rather just a single date for each event). No enrollment criteria (example: at least 6 months continuous coverage) were used when Symphony constructed the database. With these obstacles in mind, we sought to use these data to reconstruct whether a patient who tested positive for COVID-19 was admitted to the hospital because of that diagnosis. We found that researchers like us (using Symphony Health Data), overcame this hurdle in a variety of ways.

### Literature review

To uncover how researchers deal with this lack of structure in claims data, a comprehensive literature search was conducted by a health sciences librarian (E. K.). EBSCOhost Academic Search Complete, Business Source Complete, CINAHL Plus with Full Text, MEDLINE with Full Text, and OmniFile Full Text Mega (H.W. Wilson) were searched from the dates of inception through August 2021. Additionally, the search consisted of a combination of keywords and equivalent subject headings representing “Symphony Health Solutions” as a company or a reference to the use of Symphony data. The results from the Symphony search were combined with a broad variety of terms representing the concepts of classification or categorization. An English language limit was applied to results, and after deduplication, 52 articles were retrieved.

Of the 52 articles retrieved, 32 articles were deemed relevant for this study. Of the 32 remaining articles, a clear pattern was identified as to how the authors chose to reconstruct the events of interest. Eighteen studies analyzed Symphony data using one timeline [4–21] to reconstruct the event. For example, Hampp et al. [7] used the Symphony Health Solutions PHAST Prescription Monthly database to investigate the antidiabetic drug use in the US population during a predefined single timeline. Six studies looked at data with one timeline but multiple follow-ups within a time range [22–27]. Multiple timelines were used by eight of the retrieved papers [2, 28–34]. For example, Brixner et al. [2] conducted a longitudinal study using patient-level Symphony Health Solutions administrative claims data to assess the effectiveness of the HUMIRA Complete PSP in patients receiving adalimumab (ADA) treatment for broad range of diagnoses. They required the patients to have ≥ 2 claims which were at least 30 days apart to be included in the study.

Using this past work, as well as our own personal experience with claims data, we developed a generic methodology to reconstruct event specific hospitalizations. This proposed methodology is meant to act as a guide for how researchers can utilize health claims data in a more rigorous way.

## Methods

### Event reconstruction strategy

**Fig. 1:**
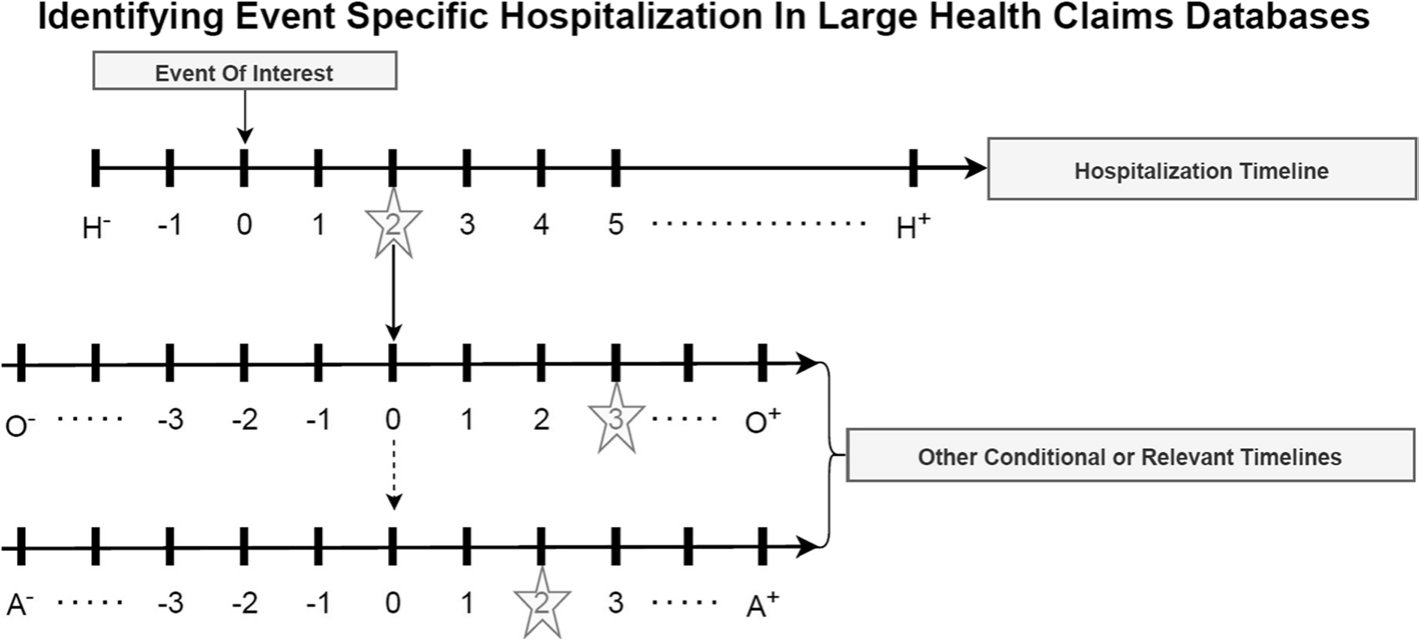
Generic event reconstruction diagram (stars indicate example scenario where example patient had a hospitalization 2 days after the event of interest and had one conditional event 3 days after their hospitalization and another conditional event 2 days after their hospitalization)

Our event reconstruction strategy centers on the overlap of various event horizons (timelines) of interest. Specifically, when it comes to identifying event specific hospitalizations, the hospitalization event horizon is an important one to define. In Fig. 1, the hospitalization.

horizon is defined as H^−^ to H^+^ where each endpoint is an integer (ℤ). Event specific hospitalizations, by definition, must have the event of interest occur within the hospitalization horizon (H^−^, H^+^). Other relevant/conditional event horizons may be defined (O^−^ to O^+^ or A^−^ to A^+^) to sensitize the definition around the time of the hospitalization. The other event horizon(s) may act as validation event(s) which may come from a contextual understanding of the problem or a literature search. If a specific patient has the event of interest within the hospitalization horizon and, if necessary, the other relevant/conditional event horizons occur within the designated horizon around the hospitalization then the patient is said to have had the event specific hospitalization. All other patients can be thought of as not having the event. In Fig. 1 (as represented by stars) an example patient had a hospitalization 2 days after the event of interest and had one conditional event 3 days after their hospitalization and another conditional event 2 days after their hospitalization. Because this patient had events within the designated horizons (H^−^ to H^+^, O^−^ to O^+^ and A^−^ to A^+^) they are said to have the event specific hospitalization.

## Results

The COVID-19 research database enables public health and policy researchers to use real-world data to better understand and combat the COVID-19 pandemic. In June 2021, via the COVID-19 research database, we gained access to the Symphony Health Data.

### Symphony data

Our symphony data had approximately 4 million patients who tested positive for COVID-19 between 03/01/2020 and 12/31/2020. While we had data after 12/31/2020, our study focused on 2020 due to access to the Food and Drug Administration (FDA) Emergency Use Authorization (EUA) COVID-19 vaccines in early 2021 and beyond which we felt would deduct from our focused study of interest. Patient records are de-identified and minimal demographic information (Age, Sex, first two digits of the patient’s residential zip code) is known about the unique patients within the dataset. Patient level CPT, diagnosis, and prescription codes were available from late 2018 to mid-2021. Using these data, which are contained in different tables accessible via the snowflake SQL platform, we utilized our event reconstruction strategy where we intended to reconstruct which, of the 4 million patients who tested positive for COVID-19, were hospitalized due to the COVID-19 diagnosis.

### Hospitalization due to COVID-19 diagnosis reconstruction

**Fig. 2:**
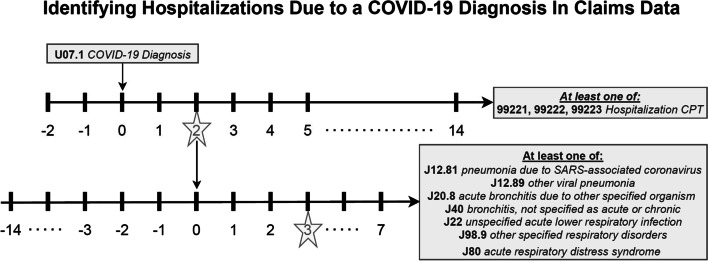
Hospitalization due to COVID-19 diagnosis reconstruction diagram (stars indicate example scenario: patient had a COVID-19 diagnosis, 2 days before they were hospitalized, and had one of the other additional diagnoses 3 days after the hospitalization then that patient would be defined as a patient who was hospitalized due to a COVID-19 diagnosis)

Using our generic definition, defined above, and outlined in Fig. 1, our clinical and research team decided the necessary event horizons endpoints. First, a hospitalization CPT code of at least one of 99,221, 99,222, or 99,223 needed to occur in the -2 to 14-day timeline from COVID diagnosis (U07.1). Within our claims data diagnoses were not ranked as they are in some claims data, so the COVID diagnosis could have been any ranked diagnosis for the specific patient (principal, secondary, …). The time lag and lead were determined as claims do not always mimic the actual timeline that the patient experienced. A sensitivity analysis of these endpoints.

showed that most of the diagnoses and hospitalizations that met our -2 to + 14 criteria actually occurred very close (-1 to + 1) to one another. If multiple hospitalization CPT codes or multiple COVID-19 diagnoses codes occurred for a specific patient, then the minimum distance between all possible combinations of diagnoses and hospitalizations were considered. As a tie breaker the earliest minimum combination which met our criteria was considered as the COVID-19 hospitalization for that specific patient. If one of the combinations met the criteria of -2 to + 14 then the patient was said to have met the first part of the criteria for being classified as hospitalized due to a COVID-19 diagnosis. As a validation, patients needed to have at least one of a set of additional diagnoses which occur around the time (-14 to + 7) of a hospitalization. This set was again, determined by our clinical and research team. These were: pneumonia due to SARS-associated coronavirus (J12.81), other viral pneumonia (J12.89), acute bronchitis due to other specified organism (J20.8), bronchitis not specified as acute or chronic (J40), unspecified acute lower respiratory infection (J22), other specified respiratory disorders (J98.9), or acute respiratory distress syndrome (J80). For example (See Fig. 2), if a patient had a COVID-19 diagnosis, 2 days before they were hospitalized, and had one of the other additional diagnoses 3 days after the hospitalization then that patient would be defined as a patient who was hospitalized due to a COVID-19 diagnosis.

### Comparison of proposed methodology and centre for disease control and prevention (CDC) estimates

The CDC provides estimates [35] of the number of symptomatic COVID-19 illness and the aggregate number of hospitalizations from February 2020 through May 2021. Unfortunately, we were unable to attain estimates for the same time period under investigation in our study (March 1^st^, 2020, through December 31^st^, 2020). Our methodology was developed independently of the CDC data, and the CDC data is meant to provide a type of external validation to our methodology.

Table 1 provides a comparison of how our methodology on the Symphony data compares to the CDC’s population estimates. Using the proposed methodology on the Symphony data, 2.7% of the patients in the age group of 18–49 years old were hospitalized due to a COVID-19 diagnosis. The CDC’s estimated that 3% of 18–49 years old were hospitalized due to symptomatic COVID-19. In the age group of 50–64 years old, 8.2% of the Symphony patients were hospitalized as compared to the 9.2% estimated by the CDC. In the age group of 65 + years old, 14.6% of the Symphony patients were hospitalized as compared to the 28.1% estimated by the CDC. Across all age groups the total percentage of Symphony patients hospitalized due to COVID-19 was 7.3% which is similar to the estimate of 7.5% by the CDC.

**Table 1:** Comparison of symphony data and CDC estimates

Age Groups	CDC Estimated COVID-19 Cases	Symphony Estimated COVID-19 Cases	CDC Estimated Hospitalizations	Symphony Estimated COVID-19 Hospitalizations	CDC Estimated Percentage of Hospitalizations (^a^)	Symphony Estimated Percentage of Hospitalizations (^b^)
18–49 years	51,581,445	1,867,749	1,533,679	49,638	3.0%	2.7%
50–64 years	17,377,602	1,045,133	1,604,612	85,271	9.2%	8.2%
65 + years	10,005,696	1,061,390	2,808,089	155,439	28.1%	14.6%
All ages	78,964,743	3,974,272	5,946,380	290,348	7.5%	7.3%

^a^ CDC Estimated Percentage of Cases = CDC Estimated COVID-19 Hospitalizations/CDC Estimated COVID-19 Cases

^b^ Symphony Estimated Percentage of Hospitalizations = Symphony Estimated Hospitalizations/Symphony Estimated COVID-19 Cases

The difference observed in the 65 + population is worrisome and warrants serious attention. Considering Medicaid claims data are not included in our Symphony data, this discrepancy is less surprising. In the United States, those who are 65 + years old qualify for Medicare and may not be privately insured. Also, those patients who are only insured by Medicaid or not insured at all would not be captured by the Symphony data. One explanation as to why so few 65 + in the Symphony data are not hospitalized due to a COVID-19 diagnosis (as compared to the CDC’s estimates) is that they may be wealthier (can afford private insurance) and are therefore healthier on average.

While our sample is older on average than the general population, our methodology’s overall estimates for the percent of COVID-19 diagnoses who were hospitalized due to a COVID-19 diagnosis is close to the CDC’s overall estimates (7.3% vs. 7.5%). The main place to compare our methodology to the CDC estimates is in the portion of the US adult population who is likely privately insured (18–64 years). Within this group, our methodology seems to capture a percent of the sample similar to that of the CDC estimates.

## Discussion

An extensive literature search identified 32 articles which sought to define events using the Symphony Health database. From these articles a clear pattern emerged. Researchers used these claims data to restructure events using one or more time horizons. This review led us to define a generic methodology which can be used by future researchers hoping to define event specific hospitalizations within their own data. In addition, this methodology can easily be adapted to be used for other diseases and medical events. Our methodology is not specific to one database and can be extended to other claims databases as well. While our attempt to validate this method did yield similar findings to the CDC’s estimates, no method is without its fair share of limitations. Currently, the Symphony Health Database are not open source or easily available without considerable cost to the researcher, which may stifle an attempt to validate this work. The inherent lack of structure and context in claims data thwart any efforts to know for certain how accurate the methodology truly is. Defining other conditional and relevant timelines is a very important step, yet there is no clear way of defining what these should be for a given event. Clinical guidance should determine the time horizon values used in defining overlapping events. Even still, these time horizon values are subject to a misunderstanding of claims data as well as systematic bias in the way claims data are recorded.

### Recommendations for replicating this work

The framework outlined in this manuscript is purposely generic so to be as useful across, what is, a diverse landscape of available claims data. We have several recommendations for replicating this framework in the readers own claims data. The researcher should first start by understanding the basic structure of the claims data available to them. For example, some claims datasets contain information batched on the claim level (what diagnoses, procedures, prescriptions happened during a timeline) or can be presented individually and not batched together at the claim level. The Symphony data available to us, for this paper, was separate non-batched data. Sometimes claims data contain what seem to be duplicate records which will need special consideration or rules on how to address them (example: remove duplicates or consider as separate events). Considering the characteristics and definition of the event of interest, the researcher may first define relevant conditional event horizons in the framework meant to validate that the event really did happen. For example, if mechanical ventilation due to COVID-19 is an event of interest, a conditional event horizon between hospitalization and mechanical ventilation may be added to Fig. 2. Any other available data (example: inpatient, admission data) that the researcher has for the patient could be used for refining the event of interest or validating the event (example: prescribed medication, or surgical procedures). Some claims data have diagnosis codes ranked (principal, secondary, …). If available, this rank can be used to strengthen the event reconstruction strategy. After identifying patients with the defined event, we recommend that the researcher compare the result with a constructed external benchmark, if available. If discrepancies exist between the framework estimates and the external benchmark this could be due to the errors in the reconstruction strategy or a bias in the available claims data. Researchers should be aware that claims data typically contain temporal aberrations which should be built into event reconstruction timelines. For example, it is possible that in the claims timeline it shows a hospital admittance on November 1st, and a COVID-19 diagnosis the following day on November 2nd. Whether these events are close enough to be considered tied together is up to the researcher and their personal event reconstruction strategy. These choices and decisions on how to construct the target event timeline, conditional/validation event horizons all impact the downstream construction of the events used for later analysis. Researchers should be transparent about this ambiguity within the limitation’s section of their corresponding academic works.

## Conclusions

In this manuscript we have defined a generic methodology to rigorously define event specific hospitalizations from claims data. Our attempt to validate this methodology vs. the CDC’s estimates showed similar estimates within those likely to be privately insured (18–64 years old). We believe this methodology will be useful for other researchers hoping to leverage large claims databases.

## Data Availability

The data that support the findings of this study are available from the COVID-19 Research Database, but restrictions apply to the availability of these data, which were used under license for the current study, and so are not publicly available. Data are however available from the authors upon reasonable request and with permission of the COVID-19 Research Database.

## Abbreviations

ICD: International Classification of Diseases
CPT: Current Procedural Terminology
ADA: Adalimumab
FDA: Food and Drug Administration
EUA: Emergency Use Authorization
CDC: Centre for Disease Control and Prevention
